# Moving toward a standardized diagnostic statement of pituitary adenoma using an information extraction model: a real-world study based on electronic medical records

**DOI:** 10.1186/s12911-022-02031-0

**Published:** 2022-12-07

**Authors:** Jingya Zhou, Xiaopeng Guo, Lian Duan, Yong Yao, Yafei Shang, Yi Wang, Bing Xing

**Affiliations:** 1 Department of Medical Records, Peking Union Medical College Hospital, Chinese Academy of Medical Sciences and Peking Union Medical College, 1 Shuaifuyuan, Dongcheng District, Beijing, 100730 China; 2 Department of Neurosurgery, Peking Union Medical College Hospital, Chinese Academy of Medical Sciences and Peking Union Medical College, 1 Shuaifuyuan, Dongcheng District, Beijing, 100730 China; 3 Key Laboratory of Endocrinology of National Health Commission, Department of Endocrinology, State Key Laboratory of Complex Severe and Rare Diseases, Peking Union Medical College Hospital, Chinese Academy of Medical Science and Peking Union Medical College, 1 Shuaifuyuan, Dongcheng District, Beijing, 100730 China; 4 Goodwill Hessian Health Technology Co., Ltd, Room 2208, 2nd Floor, Building 1, No. 7, Pioneer Road, Shangdi Information Industry Base, Haidian District, Beijing, 100085 China

**Keywords:** Pituitary adenoma, Diagnosis, Elements, Manual labeling, Automatic extraction

## Abstract

**Purpose:**

Diagnostic statements for pituitary adenomas (PAs) are complex and unstandardized. We aimed to determine the most commonly used elements contained in the statements and their combination patterns and variations in real-world clinical practice, with the ultimate goal of promoting standardized diagnostic recording and establishing an efficient element extraction process.

**Methods:**

Patient medical records from 2012 to 2020 that included PA among the first three diagnoses were included. After manually labeling the elements in the diagnostic texts, we obtained element types and training sets, according to which an information extraction model was constructed based on the word segmentation model “Jieba” to extract information contained in the remaining diagnostic texts.

**Results:**

A total of 576 different diagnostic statements from 4010 texts of 3770 medical records were enrolled in the analysis. The first ten diagnostic elements related to PA were histopathology, tumor location, endocrine status, tumor size, invasiveness, recurrence, diagnostic confirmation, Knosp grade, residual tumor, and refractoriness. The automated extraction model achieved F1-scores that reached 100% for all ten elements in the second round and 97.3–100.0% in the test set consisting of an additional 532 diagnostic texts. Tumor location, endocrine status, histopathology, and tumor size were the most commonly used elements, and diagnoses composed of the above elements were the most frequent. Endocrine status had the greatest expression variability, followed by Knosp grade. Among all the terms, the percentage of loss of tumor size was among the highest (21%). Among statements where the principal diagnoses were PAs, 18.6% did not have information on tumor size, while for those with other diagnoses, this percentage rose to 48% (*P* < 0.001).

**Conclusion:**

Standardization of the diagnostic statement for PAs is unsatisfactory in real-world clinical practice. This study could help standardize a structured pattern for PA diagnosis and establish a foundation for research-friendly, high-quality clinical information extraction.

## Introduction

Pituitary adenoma (PA), the second most common primary central nervous system (CNS) tumor, accounts for 17.1% of primary brain pathologies, with an annual incidence of 4.36 per 100,000 individuals [1]. Clinically nonfunctioning PAs present mainly with mass effects on surrounding structures, including the optic chiasm and pituitary gland, while secretory PAs also stimulate certain hypothalamic-pituitary-organ axes and present with, for instance, acromegaly and Cushing’s disease. Although the vast majority of PAs are benign and can be treated with surgery, radiotherapy, and medical therapy, some do not respond to the above therapeutic options and have a higher recurrence rate and a dismal prognosis. The individualized treatments made by multiple disciplinary teams (MDTs) have been recognized as key regimens in treating patients with aggressive, refractory PAs [2–7]. The final goals for treating PAs include tumor removal, endocrine remission, prolonged survival, and improved health-related quality of life (QoL) [8–11].

Since PAs are neuroendocrine tumors with variable, complex characteristics [12], there may be multiple irregularities and inconsistencies in the expression of diagnostic terms for this disease in the real-world clinic [13], resulting in a lack of standardization for PA diagnostic statements. However, as the core information for patients with PAs contained in electronic medical records (EMRs), accurate and standardized diagnostic statements form the basis for decision-making and are important data sources for identifying patients with various types of PAs, thus contributing to better management of the disease. In addition, structured and standardized recording of the characteristics of PAs within these diagnostic statements can not only facilitate the interaction and sharing of PA data within an MDT but also assist in the rapid access to and efficient use of those data for clinical practice and scientific research, especially for studies conducted in multiple PA register centers. However, the lack of standardization for these diagnostic statements usually results in a failure to reflect the complete characteristics of PAs, influencing the extracting of detailed information on PAs and the repeated utilization of diagnostic data and resulting in an increasing demand for standardizing the documentation of PA diagnoses among relevant specialists.

To solve the above problem, we attempted to standardize PA diagnostic statements by utilizing a Chinese word segmentation model called Jieba (14–15), implemented as a Python package, to explore the most commonly used elements contained in these statements as well as their combination patterns and variations, in real-world clinical practice. By building an optimized Jieba-based information extraction model that could be used to efficiently preprocess clinical records after loading the Medical Professional Term Dictionary into the module as the word segmentation dictionary [16], we also attempted to establish an efficient information extraction process for PA diagnostic statements. In this way, we expect that the proposed model could help doctors rapidly construct a clinical database based on the elements extracted from PA diagnostic texts. Furthermore, once the PA diagnostic statement is standardized, a structured template for the documentation of PA diagnoses could also be designed and integrated into EMR systems; thus, a convenient, efficient and standardized method for data collection, retrieval and analysis for PAs could be established in the future.

The main contributions of the study are as follows:

- Our study provides a solution for standardizing PA diagnostic statements by extracting their textual elements with an information extraction model based on the optimized Chinese word segmentation model Jieba. Furthermore, since the diagnostic texts analyzed in our study were all obtained from real-world clinics, our results could also provide a blueprint for designing structured patterns, leading to more standardized documentation for a PA diagnosis among specialists in clinical practice. This method can also be applied in standardizing diagnostic terminology for other diseases.
- Our study explores a research-friendly, high-quality clinical information extraction model developed to obtain highly detailed information from PA diagnostic statements. It could enrich the PA database simply and easily and lay a solid foundation for applying Jieba in extracting information for other diseases.

## Materials and methods

This was a retrospective study conducted at Peking Union Medical College Hospital (PUMCH). PUMCH is the China Pituitary Disease Registry Center and China Pituitary Adenoma Specialist Council and leads clinical practice and research studies in the field of MDTs for PAs in China [17–21].

### Data collection

We retrospectively collected the EMR data of patients with PAs who were admitted to our hospital from 2012 to 2020 from the institutional Electronic Medical Record Analytical Database (EMERALD). Only patients whose diagnosis of PA was among the first three diagnoses in the EMR were included. We marked the sequence for the diagnosis, with principal diagnoses marked as 1 and other diagnoses marked as 2 and 3.

### Data analysis

#### Determination of diagnostic elements and establishment of the training set

The Diagnostic Labeling Specification Team (DLST) consisted of a neurosurgeon, an endocrinologist, a medical record coordinator, and a medical record quality controller. The DLST established an annotation framework for PA discharge diagnoses and initially defined the diagnostic elements. After removing duplicate diagnosis texts, the DLST randomly selected 50 parts of the diagnostic statements and labeled them. When there were differences in the labeling results, a senior expert was invited for the final evaluation to guarantee accuracy. When manual annotation was completed in a total of 80 randomly selected parts of the diagnostic statements, all types of diagnostic elements were considered to have been fully covered. The final results of the manual annotation were then stored as a training set.

#### Selection of the chinese word segmentation model

**Fig. 1:**
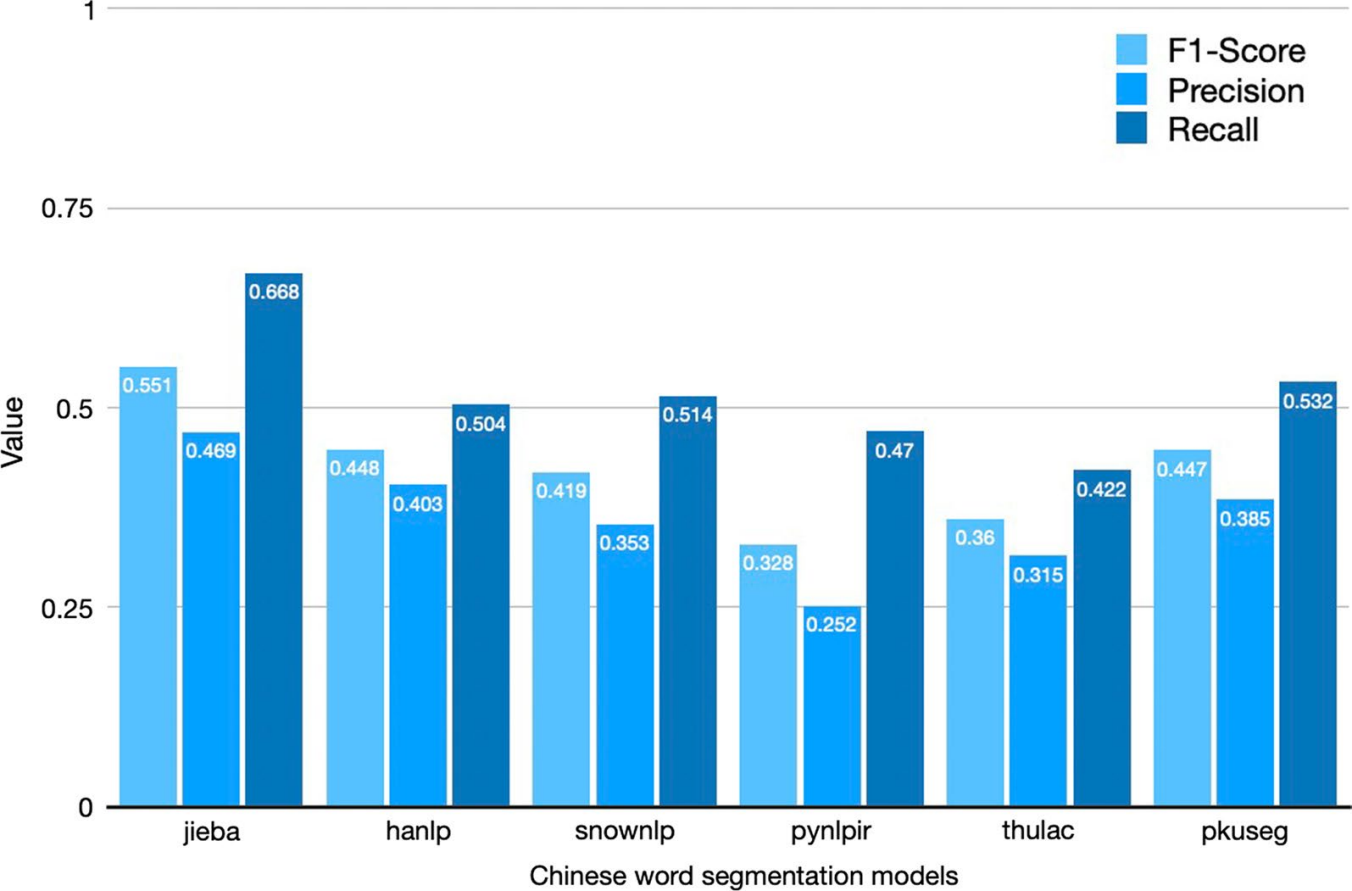
Comparison of baseline performance results for different, untrained word segmentation models. The columns with three different shades of blue respectively represent values of three parameters including F1-Score, Precision, and Recall, which were used to evaluate the performance of six word segmentation models

We enrolled different commonly used Chinese word segmentation models and compared their baseline performance parameters using the training set as the gold standard. A comparison of the performance of the different word segmentation models is shown in Fig. 1. Considering performance in terms of the accuracy of the Chinese word segmentation models, the Python package “Jieba” was finally selected as the fundamental component for word segmentation in our study. We used Jieba (http://github.com/fxsjy/jieba) to segment the PA diagnostic terms in Precise mode, one of the three modes in which Jieba can be employed, by setting the parameter “cut_all = False”. Jieba was developed based on the prefix lexicon to achieve efficient word graph scanning, generating a directed acyclic graph of all word formation cases in a sentence and then finding the maximum probability path through a dynamic planning strategy to find the maximum segmentation combination based on word frequency. For words that are not loaded into Jieba, a hidden Markov model based on the word formations of Chinese characters is used, and finally, the best word formation sequence is calculated using the Viterbi algorithm. Because Jieba does not have a named entity recognition function, in this study, we developed a multilevel medical word segmentation model with an entity recognition function based on Jieba to machine extract the diagnostic elements with a higher level of granularity, thus meeting the demands of data acquisition.

**Fig. 2:**
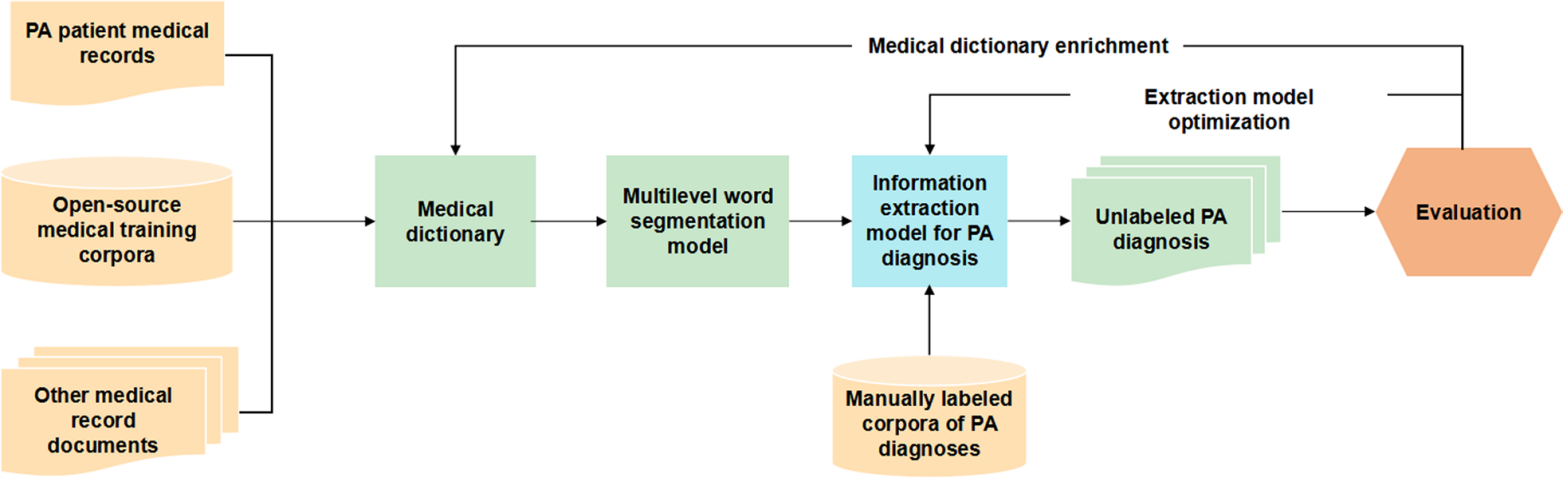
Machine extraction process for diagnostic elements. This figure illustrates the main workflow for the machine extraction of diagnostic elements

#### Machine extraction of diagnostic elements

We defined the remaining 496 diagnosis texts as the validation set and established a complete information extraction framework to accurately extract the corresponding diagnostic descriptions. The main process is outlined in Fig. 2.

**Fig. 3:**
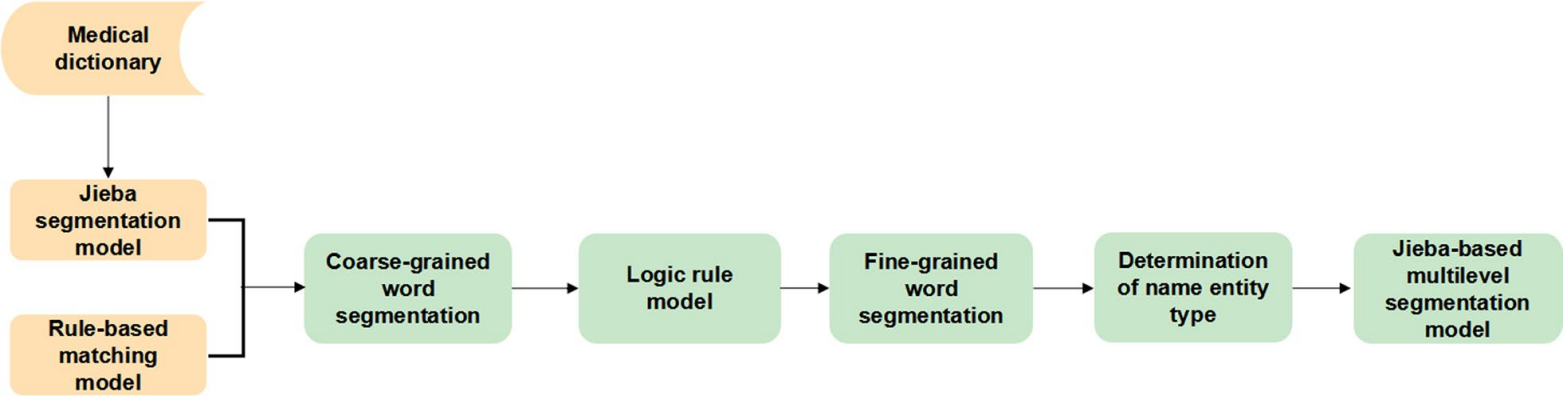
Process for constructing the Jieba-based multilevel word segmentation model. This figure specifically shows the main process for building a multilevel word segmentation model based on Jieba in order to help machine extract PA diagnostic textual elements in the following procedure

**Fig. 4:**
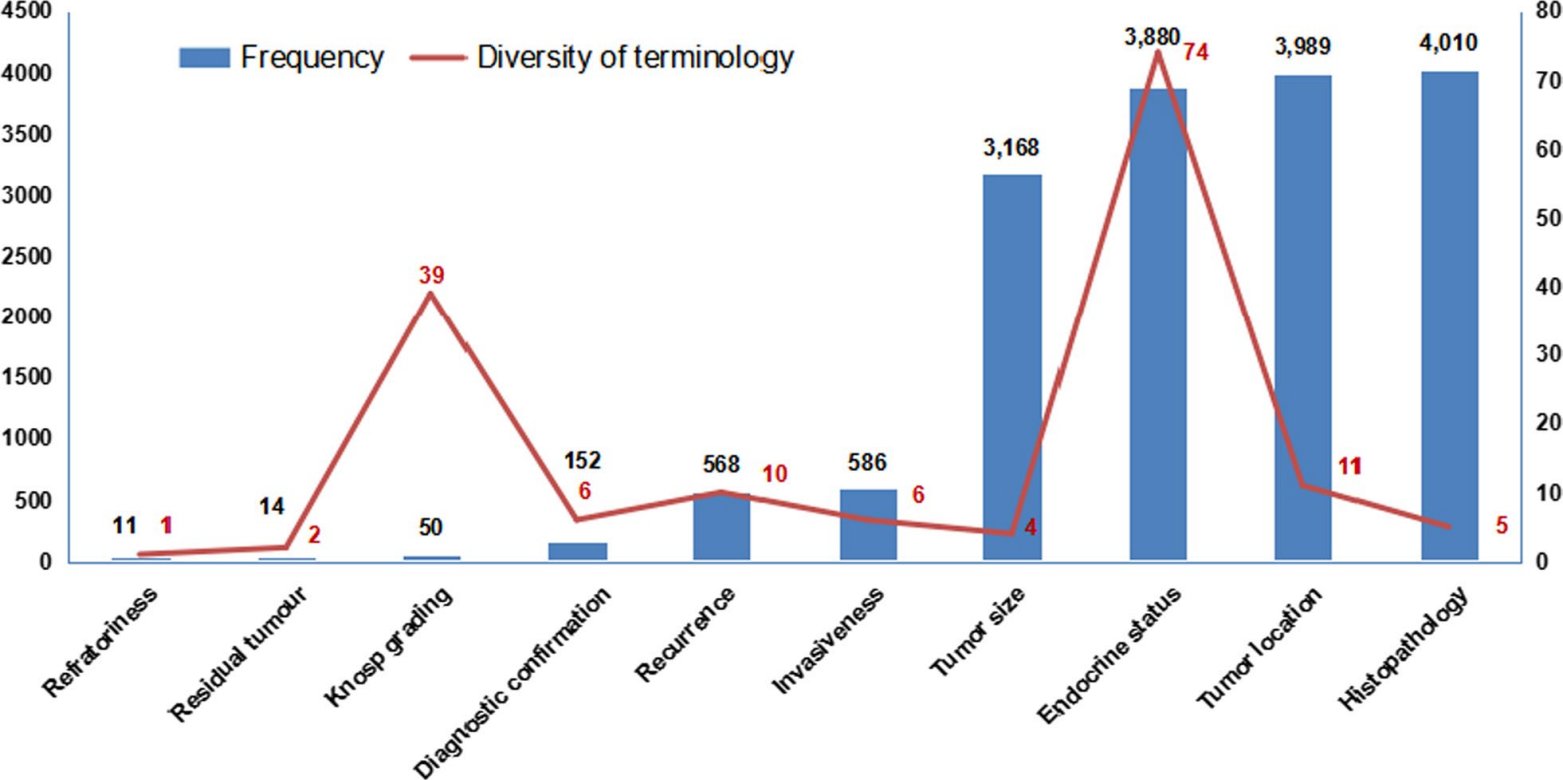
Extraction frequency and diversity of terminology for PA diagnostic elements. The blue columns show the frequency of each diagnostic element obtained by both manual labeling and machine extraction. The red curve represents the diversity of extraction terms for each diagnostic element

##### Construction process for the multilevel Jieba-based word segmentation model

Because the descriptions of the PA diagnoses were inconsistent across the reports, it was first necessary to perform word segmentation. Since it can be difficult to distinguish the word boundaries of Chinese medical terminology in the word segmentation process, in this study, we first constructed a comprehensive medical dictionary containing approximately 700,000 medical words to ensure accurate word segmentation of Jieba. These medical terms were derived from PA-related medical records, research papers, open-source medical training corpora, and other medical record documents covering various medical elements such as tumor location, disease names, endocrine status, examination names, and drug names. Furthermore, proper nouns were uploaded into Jieba to construct an improved word segmentation model for medical texts. Additional functions were developed to meet the requirements for word segmentation in our work. The Jieba package uses the directed acyclic graph method to calculate the probability path of the word segmentation results and selects the most suitable segmentation, but this leads to a long word segmentation result, thus failing to extract detailed information such as the anatomical aspect of the PA. To solve this problem, we further developed a multilevel segmentation and name entity recognition function based on Jieba. Further, considering the poor ability to recognize numbers and upper- and lowercase letters, we proposed to use the pipeline method to add a rule model and enhance the word segmentation performance. After obtaining coarse-grained word segmentation results through the previous steps, a logic rule model was added according to the extraction requirements for the PA diagnostic textual elements, and then additional fine-grained word segmentation was performed. For cases when nested entities were included in the data, we used the preliminary results of the word segmentation to perform a multilevel word segmentation process to achieve the highest level of granularity for entity recognition and meet the demand of PA diagnostic element acquisition.

For example, the diagnostic term “ACTH-secreting pituitary microadenoma” was further split by a multilevel word segmentation model, as shown in the following example: [{“word”: “ACTH-secreting pituitary microadenoma”, “entity_class”: “disease”, “value”: [{“word”: “pituitary”, “entity_class”: “organ”},{“word”: “ACTH”, “entity_class”: “medicine”},{“word”: “micro”, “entity_class”: “other”},{“word”: “adenoma”, “entity_class”: “disease”}]}] (Fig. 3).

**Fig. 5:**
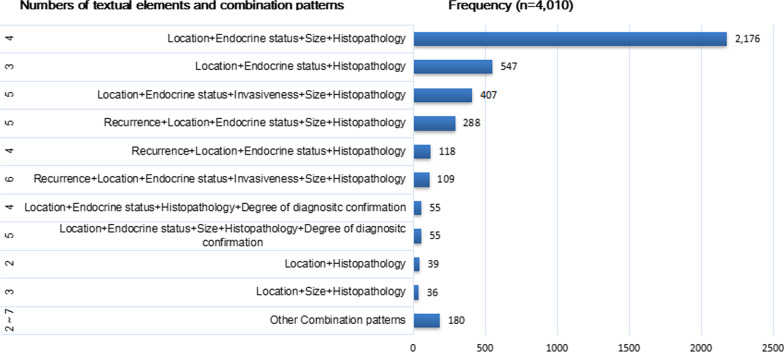
Distribution of textual element combinations of PA discharge diagnosis in the real world. The number listed in the far left represents the numbers of textual elements comprising each pattern. The horizontal blue column shows the frequency of the corresponding combination pattern. The combination patterns are described as “element1+element2+element3...”

Based on the manually annotated training set, in this study, we established an extraction model that includes multiple extraction matching patterns. If the diagnosis was described as invasive recurrent giant growth hormone secreting pituitary adenoma (Knosp grade 4 on the left, Knosp grade 0 on the right), for example, the elements were broken down as follows: recurrence, invasiveness, location, endocrine status, tumor size, histopathology, and Knosp grade. The elements of growth hormone/thyroid stimulating hormone (GH/TSH) mixed pituitary adenoma were decomposed into location, endocrine status, and histopathology. These different descriptions were automatically split through the Chinese medical word segmentation model and matched into corresponding elements according to the rules preset by the DLST.

##### Model performance evaluation and iterative optimization

The DLST reviewed the results predicted by our model from the validation set and calculated the Precision, Recall and F1-score. Problems during the review process were reflected by the DLST to the engineers. After the engineers optimized the extraction model and improved the medical dictionary, a new round of extraction was performed, and the results were returned to the DLST. To comprehensively validate the performance of the information extraction model, we further collected another 532 different discharge diagnostic texts for PA patients who were hospitalized from 2000 to 2009 from EMERALD.

### Statistical analysis

The standardized annotation was set as the gold standard. When the element information extracted by the information extraction model was consistent with the actual diagnosis description, it was called a true positive (TP); when the element information extracted by the model was inconsistent with the actual diagnosis description, it was called a false positive (FP); and when the element information contained in the diagnosis was not extracted by the model, it was called a false negative (FN). Precision (P), Recall (R) and F1-score were used to evaluate the information extraction performance. P refers to the probability that the diagnostic element information extracted was consistent with the element information that should be extracted as specified in the labeling specification and was calculated as P = TP/[TP + FP]. R refers to the probability that the element information contained in the actual diagnostic text was successfully extracted according to the labeling specification and was calculated as R = TP/[TP + FN]. The F1-score is the weighted average of precision and recall: F1 = 2*P*R/[P + R]. The extraction frequency for both the elements contained in the statement and their combination patterns were calculated based on all diagnostic texts, while the diversity of extraction for each diagnostic element was evaluated by summing the number of extracted texts after removing any duplicates; the proportion of missing text elements in the diagnostic text was calculated and compared between the principle and other diagnoses. *P* < 0.05 was considered to indicate statistical significance.

## Results

### Basic information

A total of 4084 records involving PAs at discharge were retrieved from the database, including 3770 records with diagnoses of PAs among the top three diagnoses. Finally, 4010 clinical diagnosis-free texts were included, and a total of 576 different diagnostic statements from the 4010 texts were identified after duplicates in the diagnostic texts were eliminated. Most of the records were based on information from the Department of Neurosurgery (3873/4010, 96.6%), followed by Endocrinology (82/4010, 2.1%) and others (55/4010, 1.4%).

### Determination of diagnostic textual elements

Based on the 80 manually annotated PA diagnostic texts, a total of 10 diagnostic element dimensions were summarized: tumor recurrence, tumor location, invasiveness, endocrine status, tumor size, histopathology, Knosp grade, residual tumor, diagnostic confirmation, and refractoriness. Manual annotation samples are detailed in Table 1.

**Table 1:** Manually labeled samples from PA discharge diagnostic statements covering 10 textual elements

Original diagnostic statement	Textual elements of PA discharge diagnoses									
	Tumor recurrence	Tumor location	Invasiveness	Endocrine status	Tumor size	Histopathology	Knosp grade	Residual tumor	Diagnostic confirmation	Refractoriness
Recurrent ACTH-secreting pituitary adenoma	Recurrent	Pituitary		ACTH		Adenoma				
Highly suspected aggressive nonfunctioning giant pituitary adenoma		Pituitary	Aggressive	Nonfunctioning	Giant	Adenoma			Highly suspected	
Nonfuncitoning pituitary macroadenoma (Left side Knosp grade 2–3)		Pituitary		Nonfunctioning	Macro	Adenoma	Left side Knosp grade 2–3			
Invasive nonfunctioning giant adenoma with incomplete resection		Pituitary	Invasive	Nonfunctioning	Giant	Adenoma		Incomplete resection		
Ectopic adrenocorticotropic hormone-secreting pituitary microadenoma		Ectopic pituitary		Adrenocorticotrophic hormone-secreting	Micro	Adenoma				
Refractory giant nonfunctioning pituitary adenoma		Pituitary		Nonfunctioning	Giant	Adenoma				Refractory

### Results of automatic extraction from diagnostic texts based on the 10-element scheme

The model extraction performance indicators for each element are summarized in Table 2. Problems in the extraction were mainly caused by insufficient element labeling. After manually relabeling an additional 8 PA diagnostic texts and two rounds of validation, the F1-score for each element of diagnosis reached 100%, and the model retained good performance with the test set, with F1-scores of 97.3–100.0%. Among all 4010 items identified for diagnosing PAs, the extraction frequency of the 10 elements ranged from 11 to 4010, among which tumor histopathology (4010), tumor location (3989), endocrine status (3880), and tumor size (3168) presented with the highest frequencies. In terms of expression variation, endocrine status had the largest at 74 variations, followed by Knosp grade (39 variations). However, small variations were observed for tumor location, tumor size and histopathology with respect to their frequency in the diagnostic texts. More detailed information is depicted in Fig. 4.

**Fig. 6:**
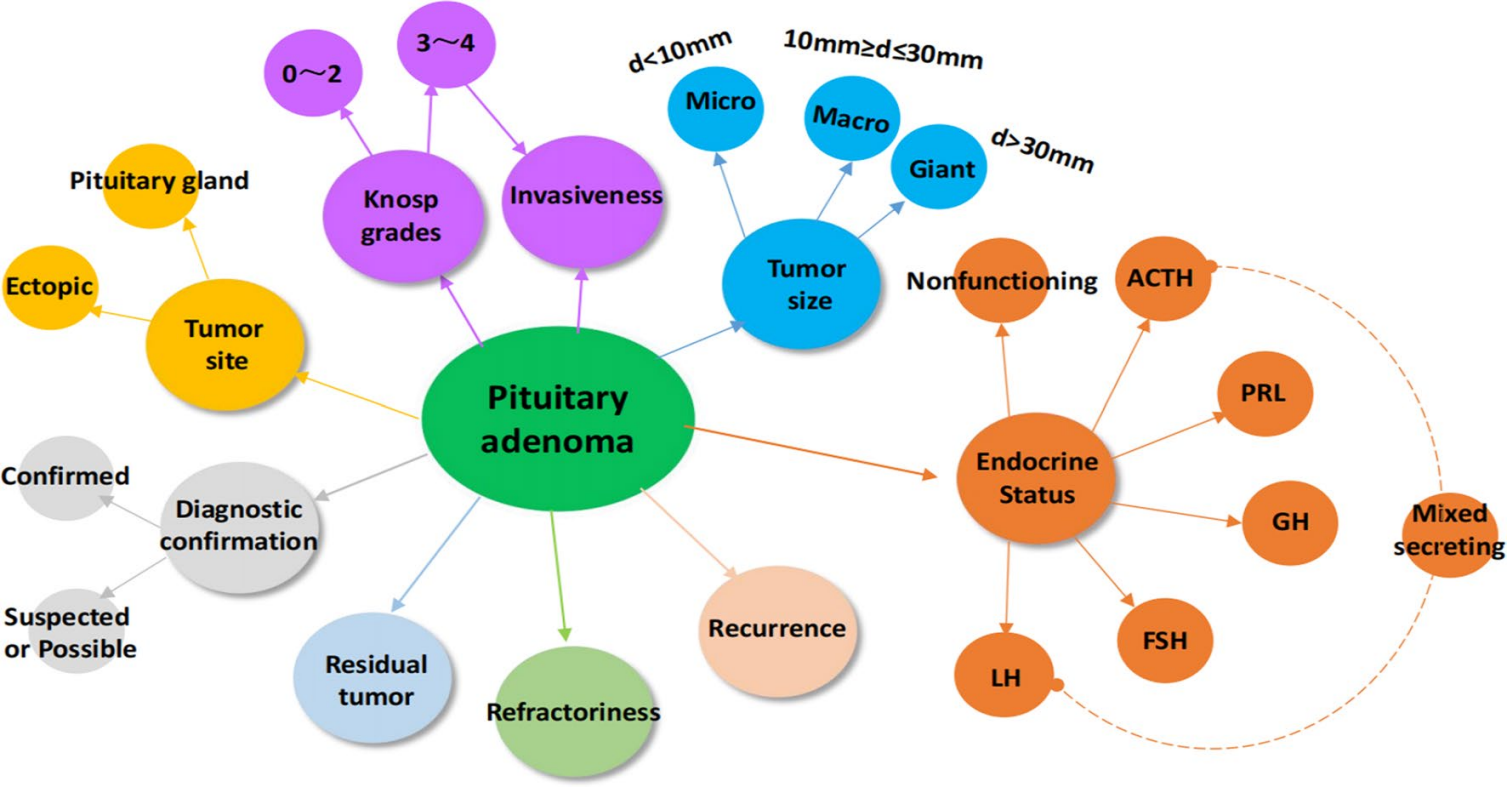
Knowledge graph of textual elements composing the PA diagnostic statements. The nine largest circles with different colors projecting from pituitary adenoma represent different diagnostic elements, and the small circles projecting from the large circles represent optional specific information that can to be considered when recording the PA diagnosis

**Table 2:** Evaluation of the diagnosis segmentation model using automatic extraction

Textual elements	First round with the validation set (n = 496)						Second round with the validation set (n = 488)						Test set (n = 532)					
	TP	FP	FN	P (%)	R (%)	F1 (%)	TP	FP	FN	P (%)	R (%)	F1 (%)	TP	FP	FN	P (%)	R (%)	F1 (%)
Tumor recurrence	134	0	0	100.0	100.0	100.0	134	0	0	100.0	100.0	100.0	98	0	0	100.0	100.0	100.0
Tumor location	477	0	4	100.0	99.2	99.6	476	0	0	100.0	100.0	100.0	531	0	1	100.0	100.0	100.0
Invasiveness	166	0	1	100.0	99.4	99.7	165	0	0	100.0	100.0	100.0	128	0	0	100.0	100.0	100.0
Endocrine status	410	0	32	100.0	92.8	96.3	437	0	0	100.0	100.0	100.0	464	1	5	99.8	98.9	99.3
Tumor size	341	0	0	100.0	100.0	100.0	338	0	0	100.0	100.0	100.0	394	0	0	100.0	100.0	100.0
Histopathology	477	0	19	100.0	96.2	98.1	488	0	0	100.0	100.0	100.0	529	0	3	100.0	99.4	99.7
Knosp grading	44	0	3	100.0	93.6	96.7	46	0	0	100.0	100.0	100.0	2	0	0	100.0	100.0	100.0
Residual tumor	4	0	0	100.0	100.0	100.0	4	0	0	100.0	100.0	100.0	8	0	0	100.0	100.0	100.0
Diagnostic confirmation	76	0	3	100.0	96.2	98.1	78	0	0	100.0	100.0	100.0	18	0	1	100.0	94.7	97.3
Refractoriness*	2	0	0	100.0	100.0	100.0	2	0	0	100.0	100.0	100.0	-	-	-	-	-	-

*No PA diagnostic statement contained textual element of refractoriness in the test set

### **Distribution of combination patterns of diagnostic elements**

A total of 2176 discharge diagnostic statements (54.3%) were composed of “tumor location”, “endocrine status”, “tumor size” and “histopathology”, representing the most popular pattern adopted by doctors for documenting PA diagnoses. The second most popular pattern applied in clinical practice was the simpler element combination of “tumor location”, “endocrine status” and “histopathology”. The third and fourth most popular patterns consisted of the most popular pattern plus the element “invasiveness” and “recurrence”, respectively. The 10 most popular combination patterns of textual elements for PA diagnoses are detailed in Fig. 5.

A knowledge graph of the textual elements composing the PA diagnostic statements was drawn based on the 10 textual elements extracted from clinical diagnostic texts in the real world and is shown in Fig. 6.

### Loss of basic elements in diagnosis

Among all the terms, the percentage of loss of tumor size was among the highest (21%). Among the principal diagnoses that were PAs, 18.6% did not have information on tumor size, while in other diagnoses, this percentage rose to 48% (Table 3).

**Table 3:** Basic diagnostic information loss [n (%)]

Element lost	Diagnosis type			*P*
	All diagnoses (n = 4010)	Principal diagnoses (N = 3685)	Other diagnoses (N = 325)	
Tumor location	21 (0.5)	5 (0.1)	16 (4.9)	< 0.001
Endocrine status	130 (3.2)	78 (2.1)	52 (16.0)	< 0.001
Tumor size	842 (21.0)	686 (18.6)	156 (48.0)	< 0.001

## Discussion

The diagnoses of PAs are complex and consist of different elements, including tumor location, size, endocrine status, and invasiveness, leading to inevitable variations in the writing of clinical diagnostic statements. In this study, we used well-trained word segmentation technology to explore the expression diversity of diagnostic texts in the medical records of PA patients in real-world clinical practice, presented commonly occurring elements that constituted the diagnostic terms for PAs, and visualized the elements through a knowledge graph. The results can not only assist young doctors in establishing a standardized diagnosis for PAs but also promote the construction of a structured diagnostic template and thus improve the efficiency of clinical information extraction and analysis. Moreover, standardization of the diagnostic statements of PAs could encourage the delivery of consistent and accurate data from multiple centers. The methods of this study can also be applied to other diseases and thereby contribute to the formation of diagnostic-treatment standards or consensuses on clinical diagnostic terminology.

This real-world-based study featured 10 elements used in the diagnosis of PAs, among which tumor location, tumor size, histopathology and endocrine status were the most used, implying sufficient focus of clinical practitioners on the above tumor characteristics, which can be included in standardizing structured PA diagnostic statements. Tumor invasiveness and recurrence status were also among the most commonly used terms, suggesting that these parameters could also be included in the diagnoses in the future.

There were approximately 600 different types of expressions for PAs among the diagnostic texts in this study, with endocrine status being the most variable. Apart from the complexity of endocrine changes in PA, the reasons for this phenomenon also include inconsistent clinical criteria in writing the diagnosis. The most highly variable elements should be given greater attention when composing the diagnostic record. English words, including “ACTH” and “Knosp”, were included in some of the diagnostic texts; spelling errors in English words contributed to the inability of the word segmentation model to accurately extract the terms.

More than one-fifth of the records lacked tumor size information. Since tumor size is an essential parameter that correlates with patient prognosis and individualized treatment [22–24], tumor size also needs to be given importance in the diagnostic statements. In the texts, more diagnoses instead of one were preferred. For example, ACTH-secreting pituitary microadenoma was in some cases written as “pituitary microadenoma” and “pituitary ACTH dependent Cushing’s syndrome”. Thus, we recommend that clinical practitioners summarize one diagnosis with the most information, and other diagnoses can be included as supplemental information.

The correctness of the ICD code in identifying a disease is related to the standardization and completeness of the clinical diagnostic description [1, 13, 25–29]. Since ICD-10 has only one code, D35.2, for describing PA, we expanded this code into several other clinical modified codes and terms, i.e., D35.202 for nonfunctioning pituitary adenoma and D35.203 for recurrent pituitary adenoma. The ICD-11 system, released in May 2018, better supports detailed clinical abstraction and comprehensive classification and better adapts to the needs of the physician in the era of mega data than the previous version [30]. The subtypes of PAs according to ICD-11 include non-secreting pituitary adenoma (2F37.0) and other secretary adenomas (2F37.Y), which more completely include detailed information on the PA, such as the endocrine status and recurrence, via postcoordination. In this way, a PA diagnosis would not be sufficiently detailed if the functional status is not annotated in the diagnosis description. Therefore, standardization of PA diagnoses is fundamental for the promotion and application of the ICD-11 in the future.

Clinicians write the medical records and are the main users of record data. It is also of great importance to accurately convert raw, free-text data into structured data that can be used to serve scientific research needs. Advances in information technology have led to the development of a variety of information extraction methods [31–35]. Studies have confirmed that the BERT-BilSTM-CRF machine learning algorithm can extract seven types of entities in medical records well (36). Although the focus of that study was on PAs, its results are generously suitable for many other diseases. Word segmentation technology can efficiently split the text information contained in a diagnostic statement according to a preset extraction element framework, for which automatic extraction is ideal. Thus, if information on different features of the PA is suitably included in the diagnostic statement, the extraction accuracy and efficiency of the extraction model will be improved.

This study has several shortcomings. First, all data were collected from a single medical center, and generalization of the results needs to be confirmed by further multicenter studies. However, the results of this study were adequately trustworthy and have high reference value since the study setting is the China Pituitary Disease Registry Center. Second, this study only extracted elements from a single, rather than multiple, diagnostic statement, and thus incomplete extraction of the overall features of PAs might have occurred. Third, given the 2022 WHO classification of PAs [36], pathology information needs to be provided at discharge to present more detailed information. However, histology data were not always available at discharge on the 2nd or 3rd postoperative day, and thus, the diagnosis at discharge did not include molecular pathological information. Fourth, to the best of our knowledge, there are no open source datasets and related algorithms for processing diagnostic textual elements similar to those used in our study; therefore, we were not able to establish benchmarks for comparing the proposed approach at the time this manuscript was written.

## Conclusion

Based on real-world medical records, we identified the elements and their combination patterns and variability used in the diagnosis of PAs by manually annotating a number of diagnostic texts and automatically extracting the segmented words, establishing a foundation for standardizing disease diagnosis template models and structured medical records for PAs and assisting in the rapid and high-quality extraction of PA information in scientific research.

## Data Availability

The data that support the findings of this study are available from the Department of Medical Records, Peking Union Medical College Hospital, but restrictions apply to the availability of these data, which were used under license for the current study and are not publicly available. Data are, however, available from the authors upon reasonable request and with permission of Peking Union Medical College Hospital.

## References

[1] Ostrom QT, Cioffi G, Waite K, Kruchko C, Barnholtz-Sloan JS (2021). CBTRUS statistical report: primary brain and other central nervous system tumors diagnosed in the United States in 2014–2018. Neurooncology.

[2] Buchfelder M, Schlaffer SM (2020). Surgical treatment of aggressive pituitary adenomas and pituitary carcinomas. Rev Endocrine Metab Disorders.

[3] Raverot G, Ilie MD, Lasolle H, Amodru V, Trouillas J, Castinetti F, Brue T (2021). Aggressive pituitary tumours and pituitary carcinomas. Nat Rev Endocrinol.

[4] Giustina A, Barkhoudarian G, Beckers A, Ben-Shlomo A, Biermasz N, Biller B, Boguszewski C, Bolanowski M, Bollerslev J, Bonert V, Bronstein MD, Buchfelder M, Casanueva F, Chanson P, Clemmons D, Fleseriu M, Formenti AM, Freda P, Gadelha M, Geer E, Gurnell M, Heaney AP, Ho KKY, Ioachimescu AG, Lamberts S, Laws E, Losa M, Maffei P, Mamelak A, Mercado M, Molitch M, Mortini P, Pereira AM, Petersenn S, Post K, Puig-Domingo M, Salvatori R, Samson SL, Shimon I, Strasburger C, Swearingen B, Trainer P, Vance ML, Wass J, Wierman ME, Yuen KCJ, Zatelli MC (2020). Melmed S. Multidisciplinary management of acromegaly: a consensus. Rev Endocrine Metab Disord.

[5] Liu X, Dai C, Feng M, Li M, Chen G, Wang R (2021). Diagnosis and treatment of refractory pituitary adenomas: a narrative review. Gland Surg.

[6] Duan L, Wang S, Zhu H, Wang R (2021). Updated key points of Chinese consensus for the diagnosis and treatment of acromegaly (2021 edition). Zhonghua Yi Xue Za Zhi.

[7] Yan JL, Chen MY, Chen YL, Chuang CC, Hsu PW, Wei KC, Chang CN (2022). Surgical outcome and evaluation of strategies in the management of growth hormone-secreting pituitary adenomas after initial transsphenoidal pituitary adenectomy failure. Front Endocrinol (Lausanne).

[8] Kasuki L, Gadelha MR (2022). Innovative therapeutics in acromegaly. Best Pract Res Clin Endocrinol Metab.

[9] Ershadinia N, Tritos NA. Diagnosis and treatment of acromegaly: an update. Mayo Clin Proc . 2022; 97**:** 333–346. 10.1016/j.mayocp.2021.11.007 35120696

[10] Castle-Kirszbaum M, Wang YY, King J, Goldschlager T (2022). Quality of life after endoscopic surgical management of pituitary adenomas. Neurosurgery.

[11] Arnardóttir S, Järås J, Burman P, Berinder K, Dahlqvist P, Erfurth EM, Höybye C, Larsson K, Ragnarsson O, Ekman B (2022). Edén Engström B. Long-term outcomes of patients with acromegaly: a report from the Swedish Pituitary Register. Eur J Endocrinol.

[12] Asa SL, Mete O, Cusimano MD, McCutcheon IE, Perry A, Yamada S, Nishioka H, Casar-Borota O, Uccella S, La Rosa S, Grossman AB, Ezzat S, Asioli S, Bozkurt SU, Comunoglu N, Cossu G, Earls P, Gazioglu N, Hickman RA, Ikeda H, Manojlovic-Gacic E, Messerer M, Öz B, Pakbaz S, Roncaroli F, Saeger W, Turchini J, Yarman S (2021). Pituitary neuroendocrine tumors: a model for neuroendocrine tumor classification. Mod Pathol.

[13] Zhou J, Zhang M, Lu L, Guo X, Gao L, Yan W, Pang H, Wang Y, Xing B (2019). Validity of discharge ICD-10 codes in detecting the etiologies of endogenous Cushing’s syndrome. Endocr Connect.

[14] Jieba project. https://github.com/fxsjy/jieba. Accessed 1 July 2022.

[15] Cao S (2020). New word detection algorithm combining correlation confidence and jieba word segmentation. Comput Syst Appl.

[16] Li L, Ayiguli A, Luan Q, Yang B, Subinuer Y, Gong H, Zulipikaer A, Xu J, Zhong X, Ren J, Zou X. Prediction and Diagnosis of respiratory disease by combining convolutional neural network and bi-directional long short-term memory methods. Front Public Health. 2022; 10**:** 881234. 10.3389/fpubh.2022.881234 PMC9114643 35602136

[17] Lian X, Shen J, Gu Z, Yan J, Sun S, Hou X, You H, Xing B, Zhu H, Shen J, Zhang F (2020). Intensity-modulated radiotherapy for pituitary somatotroph Adenomas. J Clin Endocrinol Metabolism.

[18] Zhu J, Wang Z, Zhang Y, Li X, Liu J, Deng K, Lu L, Pan H, Wang R, Yao Y, Zhu H (2020). Ectopic pituitary adenomas: clinical features, diagnostic challenges and management. Pituitary.

[19] Yang Y, Liu J, Deng K, Lu L, Zhu H, Lian X, Bao X, Duan L, Yao Y (2021). Clinical and therapeutic characteristics of pituitary TSH-secreting adenoma in adolescent-onset patients: six case studies and literature review. Front Endocrinol.

[20] Zhou J, Zhang M, Bai X, Cui S, Pang C, Lu L, Pang H, Guo X, Wang Y, Xing B. Demographic characteristics, etiology, and comorbidities of patients with cushing’s syndrome: a 10-year retrospective study at a large general hospital in China. Int J Endocrinol 2019; 2019 7159696. 10.1155/2019/7159696 PMC6399544 30915114

[21] Guo X, Zhang R, Zhang D, Wang Z, Gao L, Yao Y, Deng K, Bao X, Feng M, Xu Z, Yang Y, Lian W, Wang R, Ma W, Xing B (2022). Determinants of immediate and long-term remission after initial transsphenoidal surgery for acromegaly and outcome patterns during follow-up: a longitudinal study on 659 patients. J Neurosurg.

[22] Tang OY, Hsueh WD, Eloy JA, Liu JK (2022). Giant pituitary adenoma – special considerations. Otolaryngol Clin North Am.

[23] Chen Y, Xu X, Cao J, Jie Y, Wang L, Cai F, Chen S, Yan W, Hong Y, Zhang J, Wu Q (2022). Transsphenoidal surgery of giant pituitary adenoma: results and experience of 239 cases in a single center. Front Endocrinol (Lausanne).

[24] Micko A, Agam MS, Brunswick A, Strickland BA, Rutkowski MJ, Carmichael JD, Shiroishi MS, Zada G, Knosp E, Wolfsberger S (2021). Treatment strategies for giant pituitary adenomas in the era of endoscopic transsphenoidal surgery: a multicenter series. J Neurosurg.

[25] Mattar A, Carlston D, Sariol G, Yu T, Almustafa A, Melton GB, Ahmed A (2017). The prevalence of obesity documentation in primary care electronic medical records. Are we acknowledging the problem?. Appl Clin Inf.

[26] Asadi F, Hosseini MA, Almasi S (2022). Reliability of trauma coding with ICD-10. Chin J Traumatol.

[27] Castaldi M, McNelis J (2019). Introducing a clinical documentation specialist to improve coding and collect ability on a surgical service. J Healthc Qual.

[28] Heywood NA, Gill MD, Charlwood N, Brindle R, Kirwan CC, Allen N, Charleston P, Coe P, Cunningham J, Duff S, Forrest L, Hall C, Hassan S, Hornung B, al Jarabah M, Jones A, Mbuvi J, McLaughlin T, Nicholson J, Overton J, Rees A, Sekhar H, Smith J, Smith S, Sung N, Tarr N, Teasdale R, Wilkinson J (2016). Improving accuracy of clinical coding in surgery: collaboration is key. J Surg Res.

[29] Gologorsky Y, Knightly JJ, Lu Y, Chi JH, Groff MW (2014). Improving discharge data fidelity for use in large administrative databases. NeuroSurg Focus.

[30] Drösler SE, Weber S, Chute CG (2021). ICD-11 extension codes support detailed clinical abstraction and comprehensive classification. BMC Med Inf Decis Mak.

[31] Yang T, He Y, Yang N. Named Entity Recognition of Medical Text Based on the Deep Neural Network. J Healthcare Eng 2022;2022:3990563. 10.1155/2022/3990563 PMC8920682 35295179

[32] Tsuji S, Wen A, Takahashi N, Zhang H, Ogasawara K, Jiang G (2021). Developing a RadLex-based named entity recognition tool for mining textual radiology reports: development and performance evaluation study. J Med Internet Res.

[33] Cheng M, Xiong S, Li F, Liang P, Gao J (2021). Multi-task learning for Chinese clinical named entity recognition with external knowledge. BMC Med Inf Decis Mak.

[34] Mutinda FW, Yada S, Wakamiya S, Aramaki E (2021). Semantic textual similarity in Japanese clinical domain texts using BERT. Methods Inf Med.

[35] Fang A, Hu J, Zhao W, Feng M, Fu J, Feng S, Lou P, Ren H, Chen X (2022). Extracting clinical named entity for pituitary adenomas from Chinese electronic medical records. BMC Med Inf Decis Mak.

[36] Asa SL, Mete O, Perry A, Osamura RY (2022). Overview of the 2022 WHO classification of pituitary tumors. Endocr Pathol.

